# Randomised phase 3 study of adjuvant chemotherapy with or without nadroparin in patients with completely resected non-small-cell lung cancer: the NVALT-8 study

**DOI:** 10.1038/s41416-019-0533-3

**Published:** 2019-07-24

**Authors:** Harry J. M. Groen, Erik H. F. M. van der Heijden, Theo J. Klinkenberg, Bonne Biesma, Joachim Aerts, Ad Verhagen, Corinne Kloosterziel, Remge Pieterman, Ben van den Borne, Hans J. M. Smit, Otto Hoekstra, Frans M. N. H. Schramel, Vincent van der Noort, Harm van Tinteren, Egbert F. Smit, Anne-Marie C. Dingemans, for the NVALT Study Group, the Netherlands

**Affiliations:** 1 Department of Pulmonary Disease, University of Groningen and University Medical Center Groningen, Hanzeplein 1, Box 30.001, 9700 RB Groningen, Netherlands; 2 Department of Pulmonary Diseases, Radboud University Medical Center, Geert Grooteplein Zuid 10, 6525 GA Nijmegen, Netherlands; 3 Department of Cardiothoracic Surgery, University of Groningen and University Medical Center Groningen, Hanzeplein 1, Box 30.001, 9700 RB Groningen, Netherlands; 4 Department of Pulmonary Diseases, Jeroen Bosch Hospital, Henri Dunantstraat 1, 5223 GZ ‘s-Hertogenbosch, Netherlands; 5 Department of Pulmonary Diseases, Erasmus Medical Center, Dr Molewaterplein 40, 3015 GD Rotterdam, Netherlands; 6 Department of Cardiothoracic Surgery, Radboud University Medical Center, Geert Grooteplein Zuid 10, 6525 GA Nijmegen, Netherlands; 7 Department of Pulmonary Diseases, Isala Hospital, Dokter van Heesweg 2, 8025 AB Zwolle, Netherlands; 8 Department of Pulmonary Diseases, Ommelander Hospital Group, Pastorieweg 1, 9679 BJ Scheemda, Netherlands; 9 Department of Pulmonary Diseases, Catharina Hospital, Michelangelolaan 2, 5623 EJ Eindhoven, Netherlands; 10 Department of Pulmonary Diseases, Rijnstate Hospital, Wagnerlaan 55, 6815 AD Arnhem, Netherlands; 11 Department of Nuclear Medicine, Amsterdam University Medical Center, De Boelelaan 1117, 1081 HVAmsterdam, Netherlands; 12 Department of Pulmonary Diseases, St Antonius Hospital, Koekoekslaan 1, 3435 CM Nieuwegein, Netherlands; 13 Department of Biometrics, Antoni van Leeuwenhoek Hospital, Plesmanlaan 121, 1066 CX Amsterdam, Netherlands; 14 Department of Thoracic Oncology, Antoni van Leeuwenhoek Hospital, Plesmanlaan 121, 1066 CX Amsterdam, Netherlands; 15 Department of Pulmonary Diseases, Maastricht University Medical Center, P. Debijelaan 25, 6229 HX Maastricht, Netherlands

**Keywords:** Non-small-cell lung cancer, Non-small-cell lung cancer

## Abstract

**Background:**

Retrospective studies suggest that low molecular weight heparin may delay the development of metastasis in patients with resected NSCLC.

**Methods:**

Multicentre phase 3 study with patients with completely resected NSCLC who were randomised after surgery to receive chemotherapy with or without nadroparin. The main exclusion criteria were R1/2 and wedge/segmental resection. FDG-PET was required. The primary endpoint was recurrence-free survival (RFS).

**Results:**

Among 235 registered patients, 202 were randomised (nadroparin: *n* = 100; control *n* = 102). Slow accrual enabled a decrease in the number of patients needed from 600 to 202, providing 80% power to compare RFS with 94 events (α = 0.05; 2-sided). There were no differences in bleeding events between the two groups. The median RFS was 65.2 months (95% CI, 36—NA) in the nadroparin arm and 37.7 months (95% CI, 22.7—NA) in the control arm (HR 0.77 (95% CI, 0.53–1.13, *P* = 0.19). FDG-PET SUVmax ≥10 predicted a greater likelihood of recurrence in the first year (HR 0.48, 95% CI 0.22–0.9, *P* = 0.05).

**Conclusions:**

Adjuvant nadroparin did not improve RFS in patients with resected NSCLC. In this study, a high SUVmax predicted a greater likelihood of recurrence in the first year.

**Clinical trial registration:**

Netherlands Trial registry: NTR1250/1217.

## Background

The prognosis of patients with completely resected non-small cell lung cancer (NSCLC) is mainly determined by stage and performance status. Adding FDG-PET to CT improves not only the detection of locoregional and unexpected distant metastasis^1,2^ but also provides independent survival information based on tumour metabolic activity.^3–5^ It may identify patients who are at increased risk for recurrence and decreased survival and therefore may benefit from additional treatment.

Adjuvant chemotherapy improves overall survival (OS) in patients with completely resected NSCLC.^6–8^ The effect size of adding chemotherapy is approximately 4% at 5 years. To further improve survival, the addition of low molecular weight heparin (LMWH) may be a next step. Three major studies have indicated that the use of LMWHs may be associated with a survival benefit in cancer patients that cannot be directly linked to a reduction in venous thrombotic events (VTEs).^9–11^ However, not all studies showed a survival advantage owing to the administration of LMWH to lung cancer patients.^12^ ASCO recommendations noted a lack of sufficient data and therefore stated that anticoagulation should not be used to extend the survival of patients with cancer in the absence of other indications.^13^

We hypothesised that in patients with completely resected NSCLC with a high risk of recurrence, as defined by high FDG avidity, adding nadroparin to adjuvant chemotherapy would improve recurrence-free survival (RFS). To compare standard uptake values from PET between different participating centres, we first initiated a PET quality control programme with phantom evaluations.

## Methods

This was a prospective multicentre randomised phase 3 study with patients with completely (R0) resected stage II/III NSCLC, performance scores of 0–2, adequate organ function, and INRs <1.5 who were eligible for adjuvant chemotherapy (Supplementary Fig. 1). The exclusion criteria were wedge/segmental resection, prior chemo- or radiotherapy or contra-indication for nadroparin. The study was approved by the medical ethics committee of the University Medical Center Groningen in the Netherlands (METc nr. 2007-076). All patients provided written informed consent. Patients were randomised at the NVALT Data Center by Alea randomisation software, and the randomisation results were communicated via telephone or email; the patients were randomised after surgery to receive chemotherapy with nadroparin subcutaneously daily for 2 weeks at the therapeutic dose followed by 14 weeks at half the therapeutic dose (for a total of 16 weeks) or chemotherapy alone. Patients received four 3-week cycles of pemetrexed 500 mg/m^2^ and cisplatin 75 mg/m^2^ intravenously. In February 2009, after the enrolment of 11 patients, the protocol was amended for squamous histology; those patients received gemcitabine 1250 mg/m^2^ on day 1 and day 8 and cisplatin 75 mg/m^2^ on day 1 every 3 weeks for 4 cycles. Patients started adjuvant treatment within 6 weeks after surgery. Perioperatively, prophylactic LMWH was administered to all patients until discharge. The stratification factors were institute, WHO PS (0 or 1 vs. 2), stage, type of resection and previous malignancy.

The participating centres needed to be able to measure the standardised uptake value (SUV) in the primary tumour in a comparable way and, therefore, were obliged to adopt the NedPas protocol and be accredited by EANM Research Ltd. (EARL).^14,15^ Patients were registered for the study prior to surgery, and PET data were centrally analysed before randomisation.

The primary endpoint was RFS, which was measured from the date of randomisation to the date of first tumour relapse. The secondary endpoints were OS, dose intensity, quality of life according to the EORTC QCQ-C30/LC13, toxicity according to the CTCAE version 3.0, and health economics as measured by the EuroQol questionnaire.

### Assessments

Baseline assessments were performed after surgery with blood tests, the EuroQol questionnaire and measurements of toxicity. For staging, the 7th TNM system was used. During chemotherapy, patients were seen before every cycle. Follow-up was performed by chest X-ray every 2 months in the first 2 years after surgery and thereafter every 3 months until 5 years after surgery. Quality of life was measured at the time of randomisation and 3 weeks after the end of adjuvant chemotherapy. Health economics were measured with the EQ5D-3L questionnaire, but the results are not reported here.

### Statistics

Statistical analyses were performed with data from all eligible patients according to the intention-to-treat principle. It was estimated that RFS at 3 years after surgery + adjuvant chemotherapy would be 60% and that RFS at 3 years after surgery with adjuvant chemotherapy and nadroparin would be 75%.

Originally, the study was designed as a randomised study with patients with a high SUVmax measured in the primary tumour (Supplementary Fig. 1). In January 2010, after 60 patients were registered, the protocol was adapted due to slow accrual. SUVmax was not considered a selection criterion, and all resected patients with an indication for adjuvant chemotherapy were eligible. At that time, 60 patients were enrolled (also included in the final analysis), and the study was redesigned as a randomised study, decreasing the number of patients needed from 600 to 202, which provided 80% power to compare RFS with 94 events (α = 0.05; 2-sided log-rank test) in both arms at 3 years ranging from 60 to 75%, assuming exponential survival over 4 years of follow-up. Cox proportional hazard models were used to evaluate whether nadroparin was an independent factor affecting survival after adjustments for age, PS, stage and SUVmax. The analysis of groups with high and low SUVmax (cut off 10) values was established as a secondary endpoint.

Quality of life at 3 weeks after the end of treatment was assessed with linear models containing as independent variables the value of the quality of life measure of interest at the baseline, the treatment arm and an interaction term.

## Results

### Patients

**Fig. 1:**
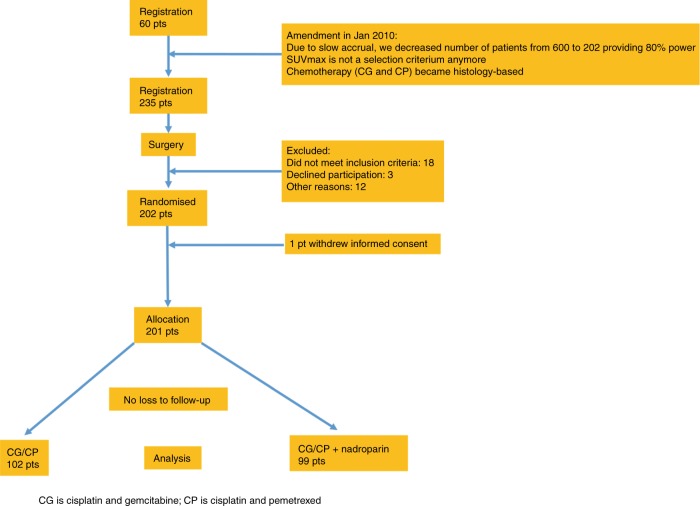
Consort diagram for the NVALT-8 study of patients registered in 15 hospitals between December 2007 and July 2013

Between December 2007 and July 2013, 235 patients were registered before surgery. A total of 202 patients were randomised, and one patient withdrew his informed consent after surgery prior to randomisation, leaving 99 patients in the nadroparin arm and 102 patients in the control arm (Fig. 1, CONSORT). Patient characteristics were similar in the two arms, except fewer patients had SUVmax values ≥10 in the nadroparin arm than in the control arm (43 vs 57%, respectively) (Table 1).

**Table 1:** Characteristics of 201 randomised patients with resected NSCLC treated with adjuvant pemetrexed or gemcitabine combined with platinum with or without nadroparin

	CP/CG	CP/CG + nadroparlne	Total
No of patients	102	99	201
Gender (M/F)	63/39	56/43	119 (59%)/82 (41%)
Age (median + range)	63 (56–69)	61 (54–67)	62 (54–69)
Performance score			
0–1	99	98	197 (98%)
2	3	1	4 (2%)
Histology			
Squamous	40 (39%)	36 (36%)	76 (38%)
Non-squamous	62 (61%)	63 (64%)	125 (62%)
TNM stage			
pT1N1	32 (31%)	27 (27%)	59 (29%)
pT2N0	6 (6%)	3 (3%)	9 (4%)
pT2N1	24 (24%)	27 (27%)	51 (25%)
pT3N0	17 (17%)	22 (22%)	39 (19%)
pT1-4N0-2 (stage IIIA)	21 (21%)	18 (18%)	39 (19%)
pT1-4N1-3 (stage IIIB)	2 (2%)	2 (2%)	4 (2%)
FDG-PET. O=D			
SUVmax <10	20	33	53 (26%)
SUVmax ≥10	75	57	132 (66%)
NEDPAS or EARL not fulfilled	8	8	16 (8%)
Surgery			
(Bi)lobectomy	81 (79%)	77 (78%)	158 (79%)
Pneumonectomy	20 (20 %)	22 (22%)	42 (21%)
Other	1 (1%)	0 (0%)	1 (<1%)
R0	98 (95%)	94 (96%)	192 (96%)
R1	4 (4%)	5 (5%)	9 (4%)
Time from surgery to start chemo (wk)	5 (5–6)	5 (5–6)	5 (5–6)

### Surgery and adjuvant treatment

The surgical resections were pathologically complete (R0) in 96% of the patients. Subsequently, 69% of the patients completed 4 cycles of adjuvant chemotherapy (nadroparin arm, 64%; control, 74%). The mean dose intensity for platinum chemotherapy with and without nadroparin was 92% and 91%, respectively; the nadroparin dose intensity was 84%. Ninety-nine percent of patients received full-dose nadroparin for 2 weeks, 92 patients received the medication during the half-dose period, 2 patients needed a dose reduction, and 5 patients had poor documentation of the dosing.

### Toxicity

No differences in toxicities, especially bleeding events, were observed between the arms, except for grade ≥3 toxicity neutropenia, which was more common in the nadroparin arm (20 vs 6 patients, *P* = 0.002).

### Recurrence-free survival

**Fig. 2:**
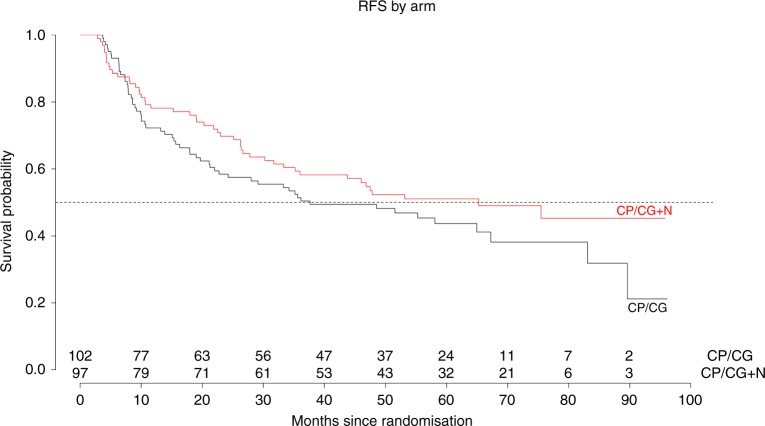
Recurrence-free survival in patients with resected NSCLC treated with adjuvant chemotherapy with or without nadroparin. CP/CG +N is cisplatin and pemetrexed/cisplatin and gemcitabine + nadroparin. HR was 0.77 (95% CI., 0.53–1.13, *P* = 0.19)

**Fig. 3:**
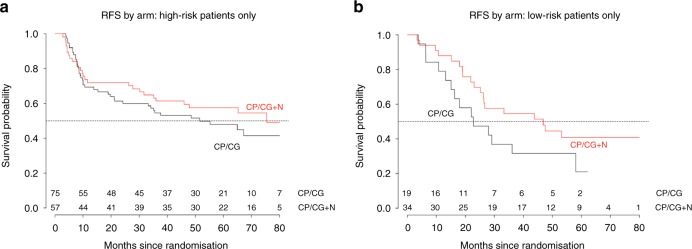
**a**/**b** Recurrence-free survival in patients with resected NSCLC treated with adjuvant chemotherapy with or without nadroparin stratified into SUVmax-based risk groups. High risk is defined as SUVmax ≥10 in the primary tumour, while the low risk is defined as SUVmax <10. For FDG-based risk groups, the HR was 0.70 (95% CI., 0.46–1.04, *P* = 0.08)

On October 26, 2017, the database was locked after 107 RFS events, with 48 in the nadroparin arm and 59 in the control arm. The median follow-up period was 63.1 months (95% CI, 60.5–68.4). The median RFS was 65.2 months (95% CI, 36—NA) in the nadroparin arm and 37.7 months (95% CI, 22.7—NA) in the control arm (HR 0.77, 95% CI, 0.53–1.13, *P* = 0.19) (Fig. 2). The 3-year RFS was 59% (95% CI, 50–70) in the nadroparin arm and 51% (95% CI, 42–62) in the control arm. After stratification into FDG-based risk groups, the hazard ratio was 0.70 (95% CI, 0.46–1.04, *P* = 0.08) (Fig. 3a, b).

### Overall survival

**Fig. 4:**
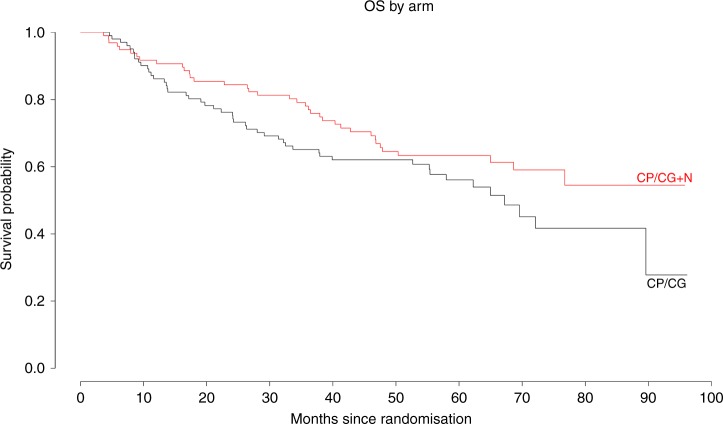
Overall survival in patients with resected NSCLC stratified by treatment arm. CP/CG +N is cisplatin and pemetrexed/cisplatin and gemcitabine + nadroparin. The HR was 0.70 (95% CI 0.46–1.08, *P* = 0.10)

Overall survival was not different between the two arms. Overall, 85 patients died; 37 patients in the nadroparin arm died, and 48 patients in the control arm died (HR 0.70 (95% CI., 0.46–1.08), *P* = 0.10) (Fig. 4). After stratification into FDG-based risk groups, the HR became 0.67 (95% CI 0.42–1.05, *P* = 0.08) in the univariate analysis and 0.75 (95% CI., 0.47–1.2, *P* = 0.24) in the multivariate analysis. The 3-year survival rates were 78% (95% CI, 56–75) in the nadroparin arm and 65% (95% CI, 56–75) in the control arm.

### Metabolic tumour activity measured by FDG-PET

The median SUVmax value of the primary tumour in 185 baseline scans was 13.7, ranging from 9.2 to 18.4; the median values were not different between the two arms. Eight percent of the scans (*n* = 16) did not fulfil the NEDPAS criteria or the centres were not accredited by EANM Research Ltd. (EARL) (Table 2). Of note, the phantom calibration of PET scans is part of the EARL accreditation requirements. The local and central SUVmax measurements were 97% congruent for the cut-off value of 10. In the first year after randomisation, RFS was inferior for patients with high SUVmax values (HR 0.48, 95% CI 0.22–0.9, *P* = 0.05) (Supplementary Fig. 2).

### Quality of life

The overall quality of life was not different between the treatment groups. Quality of life modelling revealed that peripheral neuropathy and, to a lesser extent, cognitive functioning were statistically associated with nadroparin treatment after chemotherapy. This was not the primary outcome, so this observation should be confirmed in larger studies.

## Discussion

Originally, the study was designed to test the hypothesis that in patients with resected NSCLC and a high risk of recurrence (FDG SUVmax ≥10), adding nadroparin to adjuvant chemotherapy would improve their recurrence-free survival. The study accrued participants slowly and therefore was redesigned. In this study, adjuvant nadroparin in patients with resected NSCLC undergoing chemotherapy did not improve RFS (HR 0.77, *P* = 0.19) after stratifying for FDG avidity in the primary tumour (HR = 0.70, *P* = 0.08). No difference in RFS of 15% or larger was observed in this study; we cannot exclude the possibility of a smaller difference in RFS. Additionally, the survival curves do not cross throughout their course. Blocking circulatory tumour cells from invading different organs with currently available drugs such as bevacizumab or tinzaparin as adjuvant treatment is not a major method of improving survival in patients with early-stage NSCLC.^16,17^ The reason for our study result may be a lack of power and the opposing influence of the metabolic correction of SUVmax values after 1 year, leading to crossing survival curves at 20 months. In our study, we observed two different patient groups in the high SUVmax group, one with a very short survival time and one with a longer survival time (Supplementary Fig. 2).

A recent meta-analysis of the prognostic value of FDG-PET/CT in surgical NSCLC patients included 36 studies with 5807 patients.^18^ It was shown that a high SUVmax predicted a higher risk of recurrence and death and therefore can be used for risk stratification for disease control and survival. The negative prognostic role of high FDG uptake remained similar in the analyses stratified according to stage, pathology and FDG cut-off values. Our study shows that such a detrimental effect of high metabolism in the primary tumour disappears after 1 year, irrespective of nadroparin use.

The different biological functions of LMWH, the impairment of the occurrence of metastases by the inhibition of tumour cell growth by heparin-binding growth factors, tumour cell invasion by heparin-inhibition enzyme systems, tumour cell metastasis by heparin-binding cell surface selectins, tumour angiogenesis, and tumour matrix formation were, together with the inconclusive results from previous studies, the impetus and rationale for performing this study.^19–21^ A previous study by Meyer et al.^17^ examined the effects on survival of tinzaparin and did not find any benefit for early-stage NSCLC patients. Therefore, low molecular weight heparins, nadroparin and tinzaparin are not recommended as adjuvant anti-metastatic agents for patients with early-stage NSCLC.

In conclusion, adjuvant nadroparin in patients with resected NSCLC undergoing adjuvant chemotherapy did not improve RFS even after adjusting for the metabolic activity of the primary tumour. A high SUVmax value in the primary tumour predicts a worse recurrence-free survival in resectable NSCLC in the first year but not thereafter.

## Supplementary information

Supplementary files

## Data Availability

The dataset used and analysed in the current study is available from the corresponding author on reasonable request. All data are stored at the NVALT Data Center at the National Cancer Center Netherlands.
