# Supplementary material for: Randomised phase 3 study of adjuvant chemotherapy with or without nadroparin in patients with completely resected non-small-cell lung cancer: the NVALT-8 study
Source: Br J Cancer. 2019 Jul 24;121(5):372–7. doi: 10.1038/s41416-019-0533-3 (PMC6738047; doi:10.1038/s41416-019-0533-3)
Supplement: Supplementary file 1 — Supplementary files [file 41416_2019_533_MOESM1_ESM.docx]

**Supplementary Figures and Legends**

**
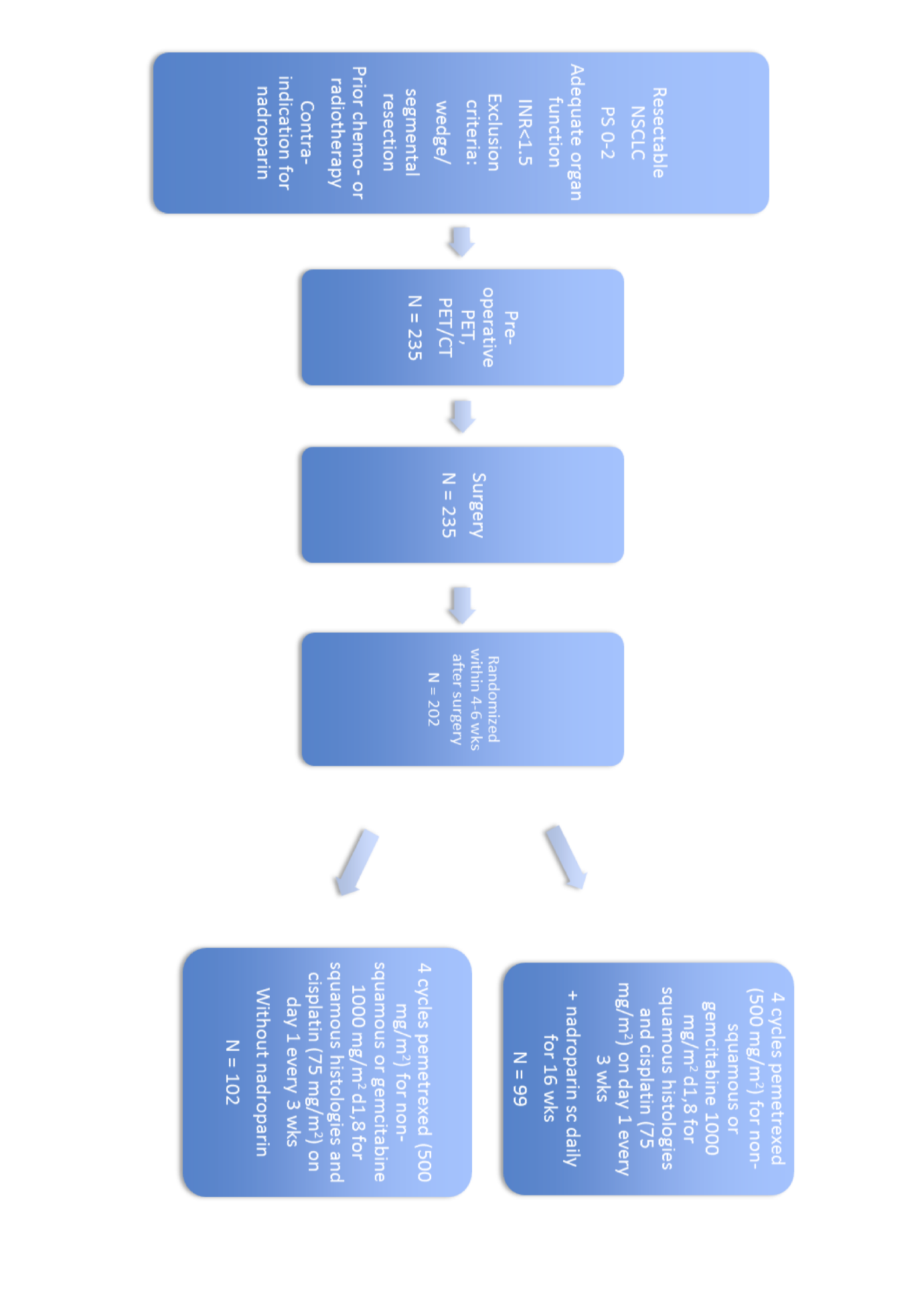
**

**Figure 1.** (only available on-line): NVALT-8 study design.

**
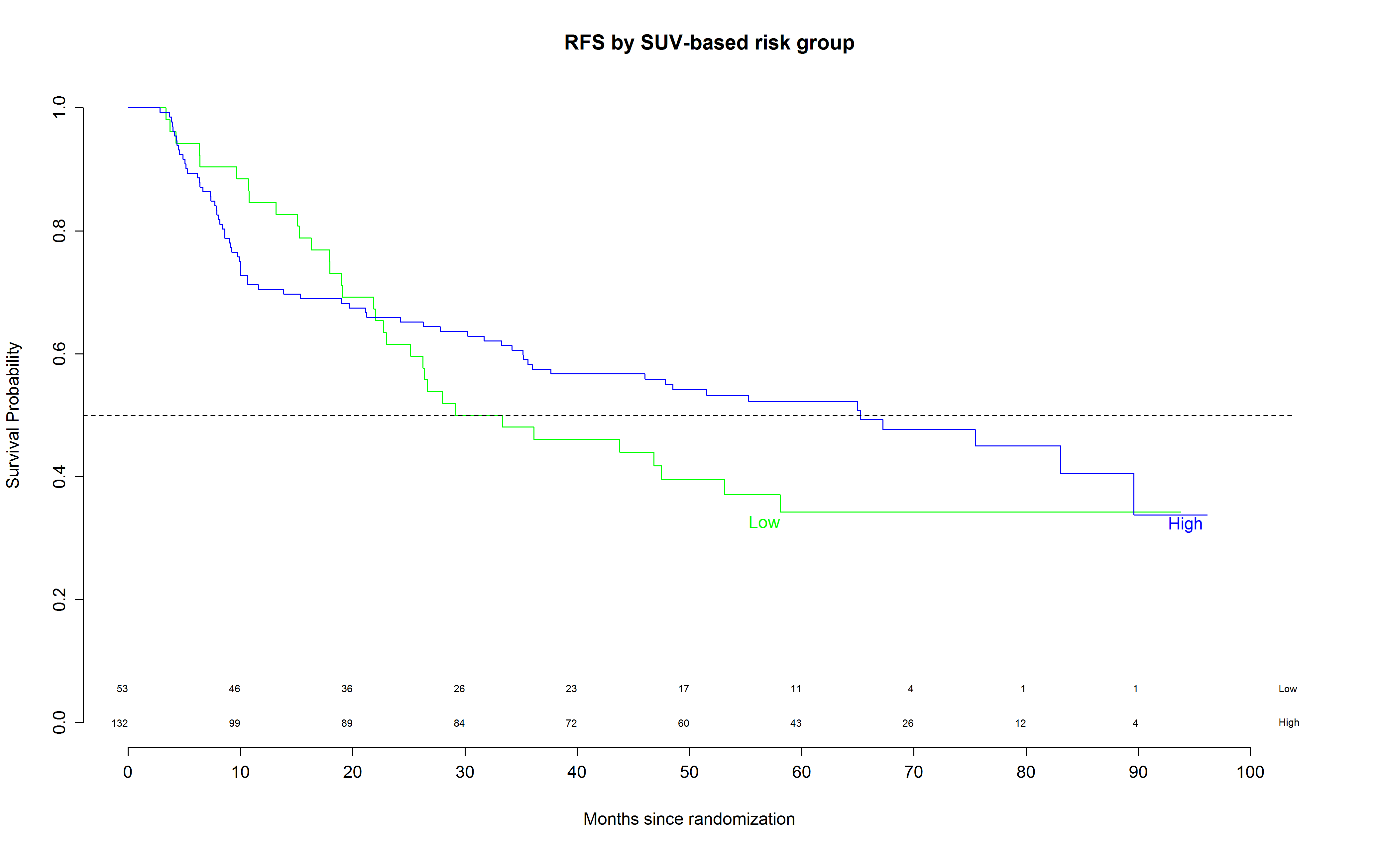
**

**Figure 2** (only available on line)**.** Recurrence-free survival in patients with resectable NSCLC stratified into SUVmax-based risk groups.

High risk was defined as SUVmax ≥ 10 in the primary tumour, while low risk was defined as SUVmax < 10. Patients with high versus low SUVmax values had different recurrence-free survival rates in the first year (HR 0.48, 95% CI 0.22 – 0.9, *P* = 0.05); thereafter, there were no differences. Crossing of the curves occurred at 20 months.
